# Supplementary material for: Targeting the transcription factor HES1 by L-menthol restores protein phosphatase 6 in keratinocytes in models of psoriasis
Source: Nat Commun. 2022 Dec 19;13:7815. doi: 10.1038/s41467-022-35565-y (PMC9763329; doi:10.1038/s41467-022-35565-y)
Supplement: Supplementary file 1 — Supplementary information [file 41467_2022_35565_MOESM1_ESM.pdf]

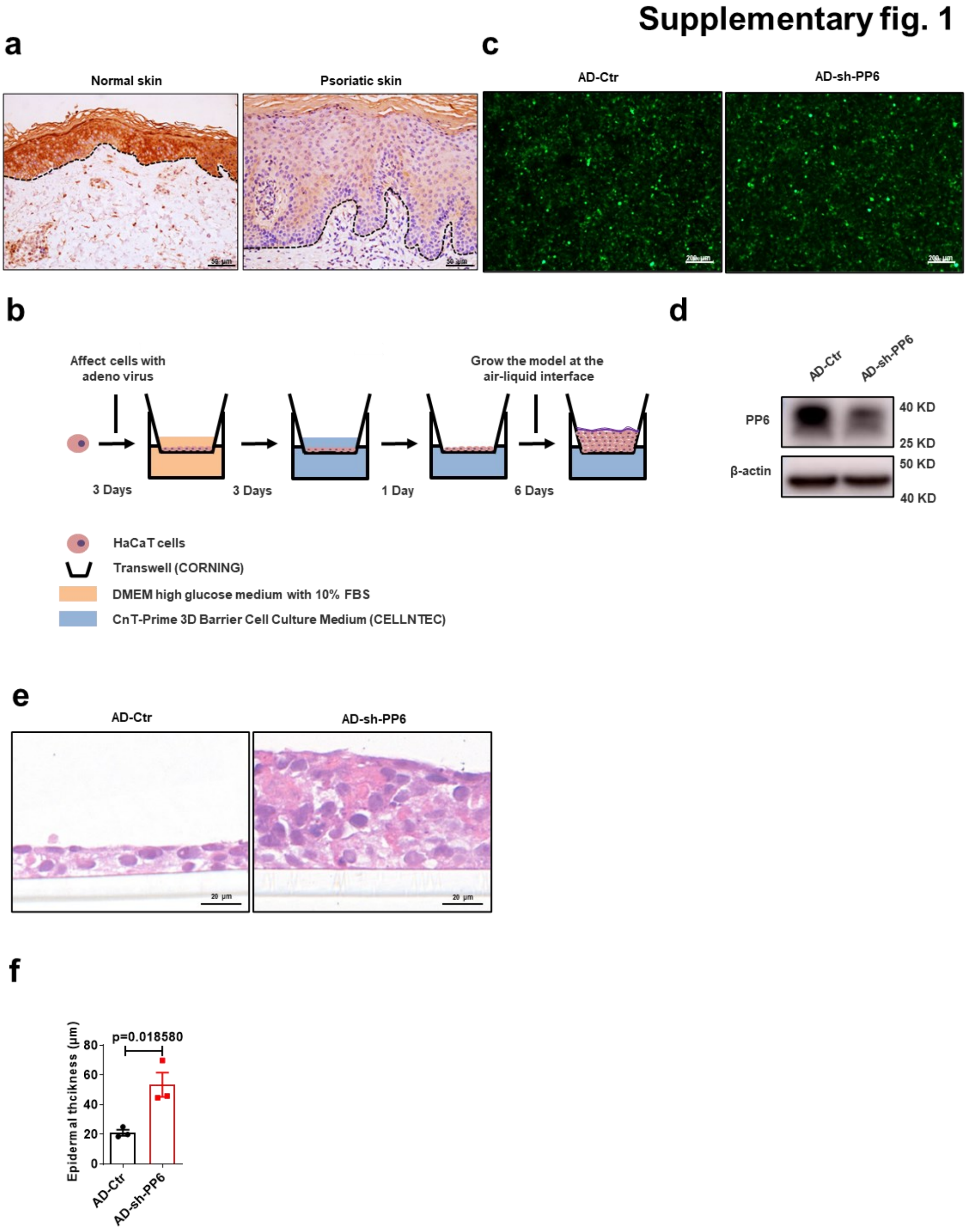

Supplementary fig. 1 PP6 down-regulation in keratinocytes increases epidermal thickness in 3-D human skin equivalent.

a, Immunohistochemical staining of PP6 in skin sections derived from healthy donors and patients with psoriasis. Scale bar, 50 μm. **b**, Schematic of establishing 3-D human skin equivalent with GFP-expressing adeno virus (AD) affected HaCaT cells. **c,** Fluorescence images showing GFP signals of

the HaCaT cells 3 days after the affection of adeno virus. Scale bar, 200 μm. **d,** Immunoblotting analysis of PP6 expression in HaCaT cells 3 days after the affection of GFP-expressing Adeno virus. **e,** Representative hematoxylin and eosin (H&E) staining of 3-D human skin equivalent established with adeno virus affected HaCaT cells. Scale bar, 20 μm. **f,** Epidermal thickness of 3-D human skin equivalent established with adeno virus affected HaCaT cells (n=3). The data in (**a, c-f**) are representative of three independent experiments. Statistical analyses were performed by two-tailed Student's t test. All data are presented as mean values ± SEM. Specific p-values are indicated in the figure. Source data are provided as a Source Data file.

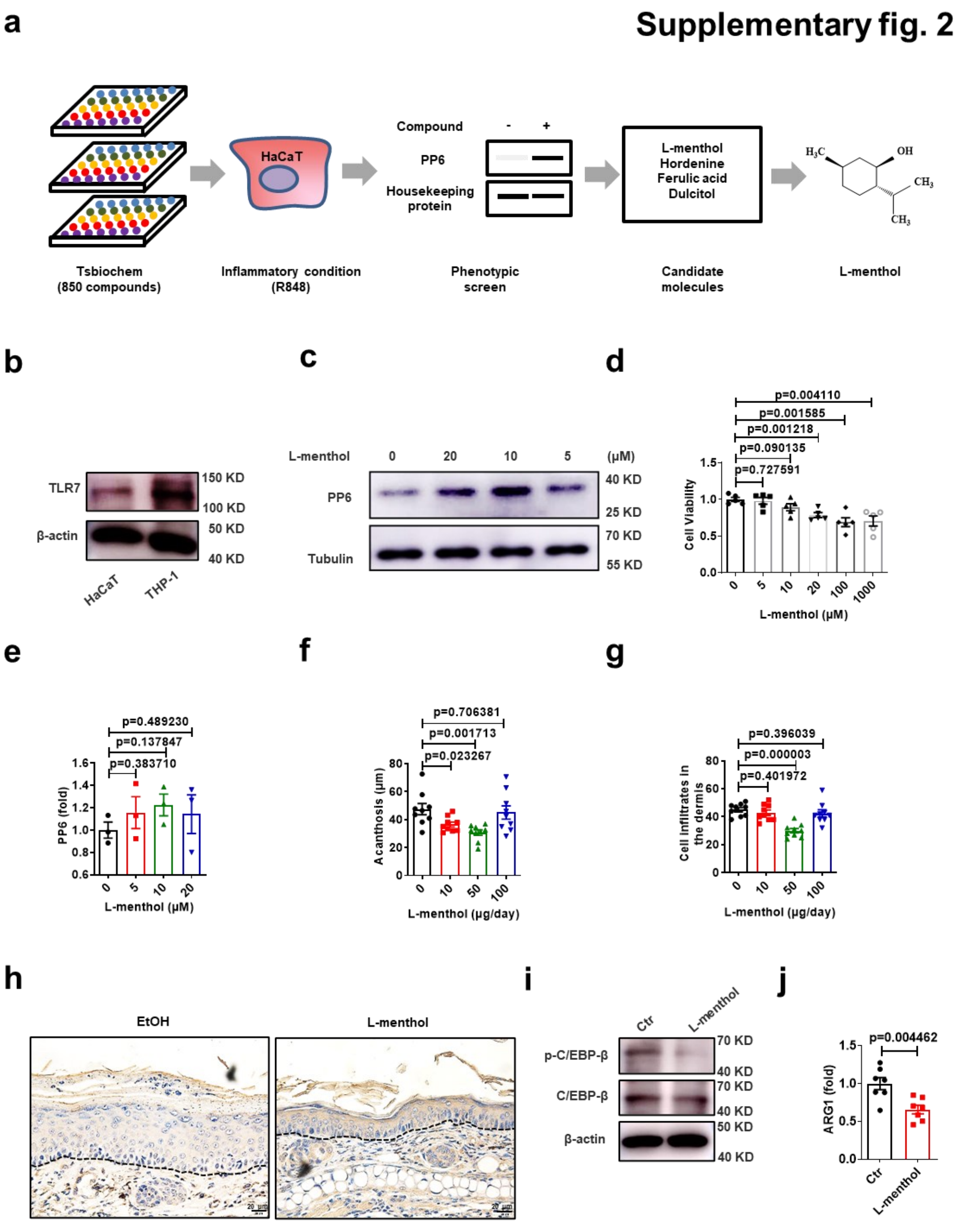

Supplementary fig. 2 Identification of the PP6-upregulating compound.

a, Schematic of screening for the PP6-upregulating compound. **b,** Immunoblotting of TLR-7 expression in HaCaT cells and THP-1 cells. **c,** Immunoblotting analysis of PP6 expression in HaCaT cells with or without L-menthol treatment for 12 h in the presence of 1 μg/ml R848. **d,** Cell viability of HaCaT cells treated with 0, 5, 10, 20, 100, and 1000 μM L-menthol for 12 hours in the presence

of 1 μg/ml R848. **e,** PP6 mRNA in HaCaT cells treated with 0, 5, 10, and 20 μM L-menthol for 12 h in the presence of 1 μg/ml R848. Results are presented as the ratio of PP6 to the GAPDH, relative to that in control (n=3). **f-g,** Acanthosis (**f**) and dermal cellular infiltrates (**g**) of ears from IMQ induced mice treated with EtOH or L-menthol (n=9). **h,** Representative immunohistochemical staining of Pp6 in ears derived from EtOH or 50 μg/day L-menthol-treated IMQ-induced mice. Scale bar, 20μm. **i,** Immunoblotting analysis of p-C/EBP-β, C/EBP-β and PP6 expression in HaCaT cells with or without 10 μM L-menthol treatment for 12 h in the presence of 1 μg/ml R848. **j,** ARG1 mRNA in HaCaT cells treated with or without 10 μM L-menthol for 12 h in the presence of 1 μg/ml R848. Results are presented as the ratio of ARG1 to the GAPDH, relative to that in control (n=7). The data in (**b-j**) are representative of three independent experiments. Statistical analyses were performed by two-tailed Student's t test. All data are presented as mean values ± SEM. Specific p-values are indicated in the figure. Source data are provided as a Source Data file.

Supplementary fig. 3

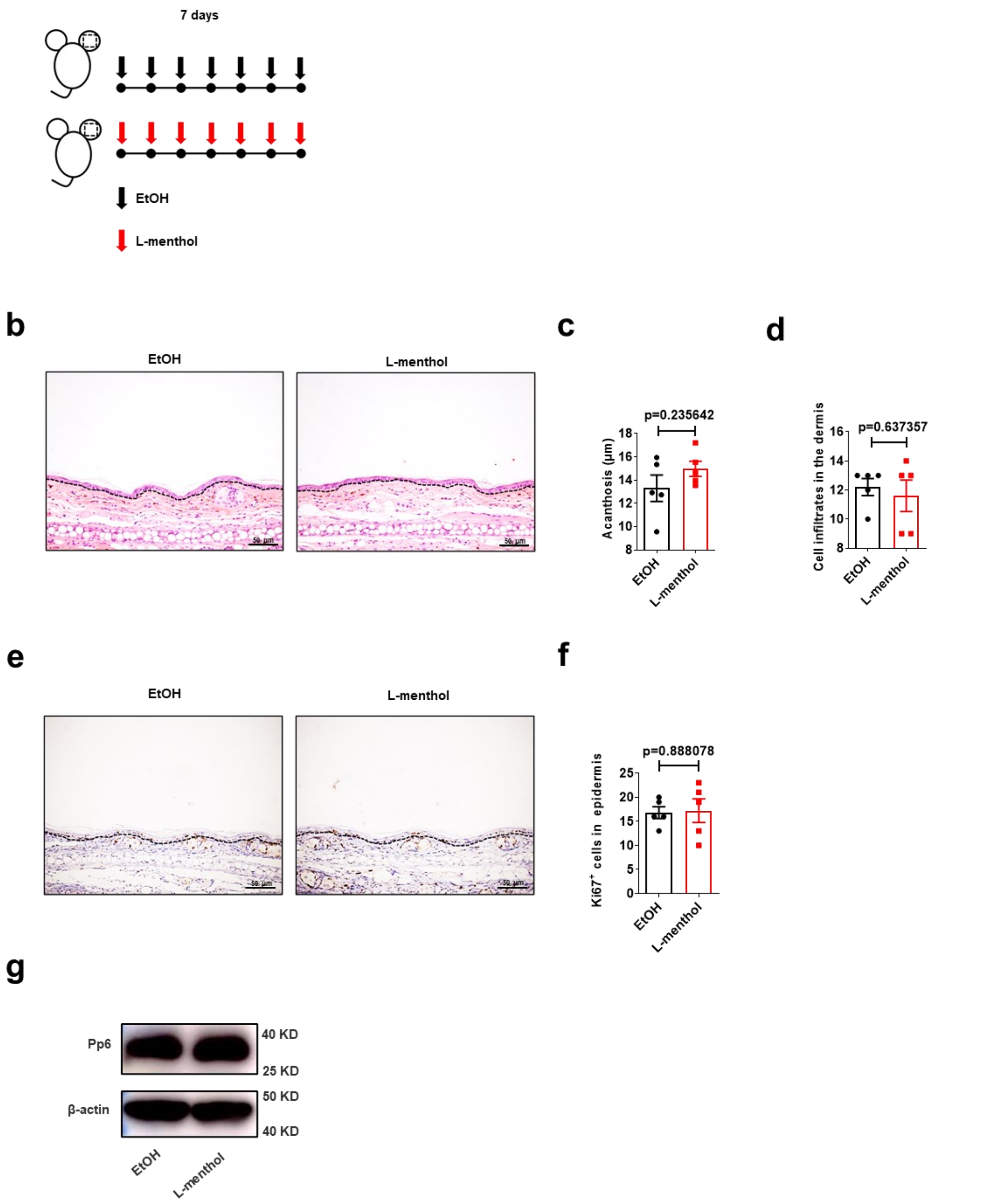

Supplementary fig. 3 L-menthol does not affect skin homeostasis under the normal condition. **a,** Schematic of 50 μg/day L-menthol treatment. **b,** Representative H&E staining of the ears of EtOH or 50 μg/day L-menthol-treated mice. Scale bar, 50 μm. **c-d,** Acanthosis (**c**) and dermal cellular infiltrates (**d**) of ears treated as in (**b**) (n=5). **e,** Representative immunohistochemical staining of Ki67 of ears treated as in (**b**). Scale bar, 50 μm. **f,** Quantitation of $Ki67^+$ epidermal cells in ears

treated as in (**b**) (n=5). **g,** Immunoblotting of Pp6 expression in epidermis derived from EtOH or 50 μg/day L-menthol-treated mice. The data in (**b-g**) are representative of three independent experiments. Statistical analyses were performed by two-tailed Student's t test. All data are presented as mean values ± SEM. Specific p-values are indicated in the figure. Source data are provided as a Source Data file.

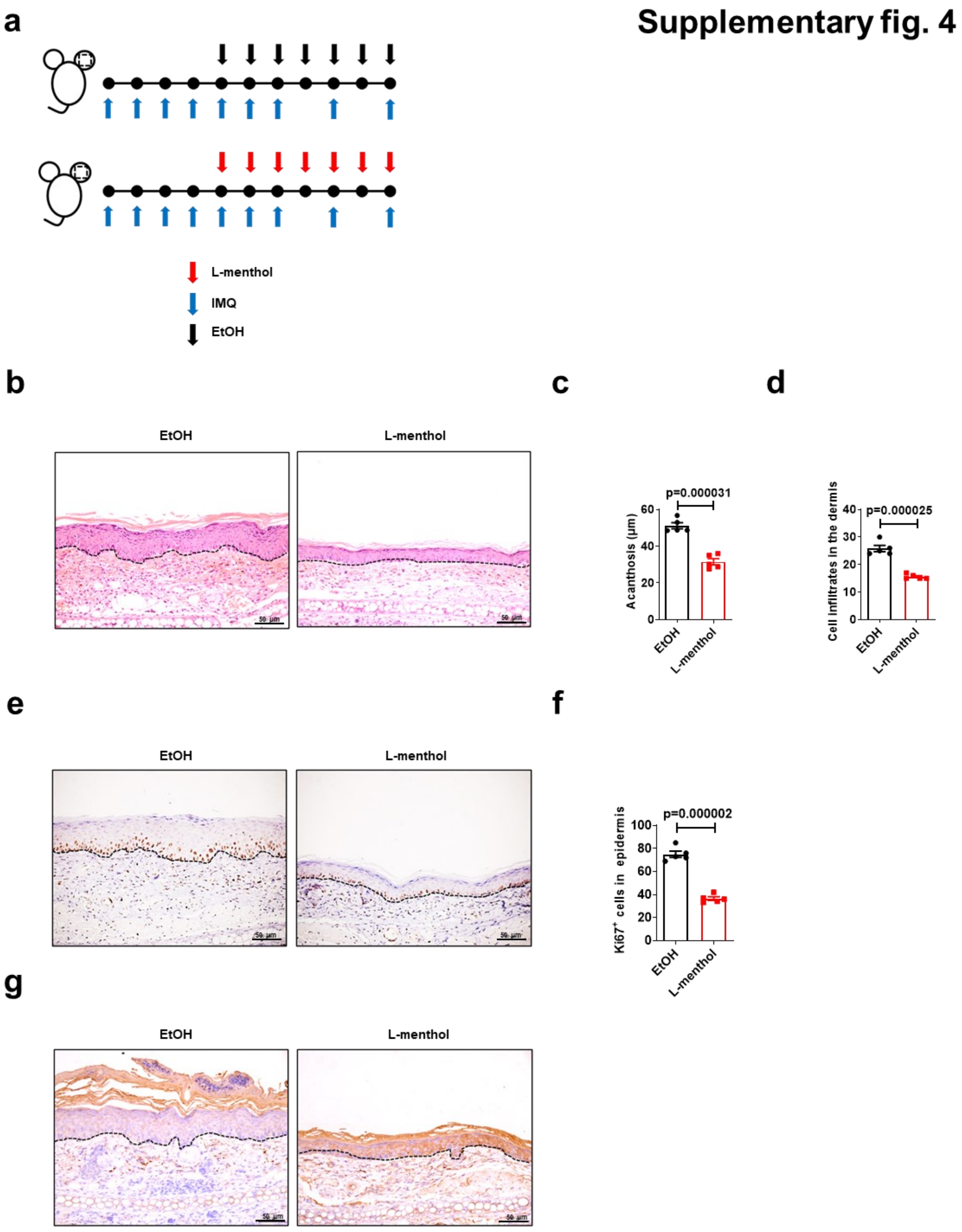

Supplementary fig. 4 L-menthol relieves psoriasis-like skin inflammation in mice.

a, Schematic of IMQ-induced psoriasis mouse model and 50 μg/day L-menthol treatment. **b,** Representative H&E staining of the ears of EtOH or 50 μg/day L-menthol-treated IMQ-induced mice. Scale bar, 50 μm. **c-d,** Acanthosis (**c**) and dermal cellular infiltrates (**d**) of ears treated as in (**b**) (n=5). **e,** Representative immunohistochemical staining of Ki67 of ears treated as in (**b**). Scale

bar, 50 μm. **f,** Quantitation of $Ki67^+$ epidermal cells in ears treated as in (**b**) (n=5). **g,** Representative immunohistochemical staining of Pp6 in ears treated as in (**b**). Scale bar, 50μm. The data in (**b-g**) are representative of three independent experiments. Statistical analyses were performed by two-tailed Student's t test. All data are presented as mean values ± SEM. Specific p-values are indicated in the figure. Source data are provided as a Source Data file.

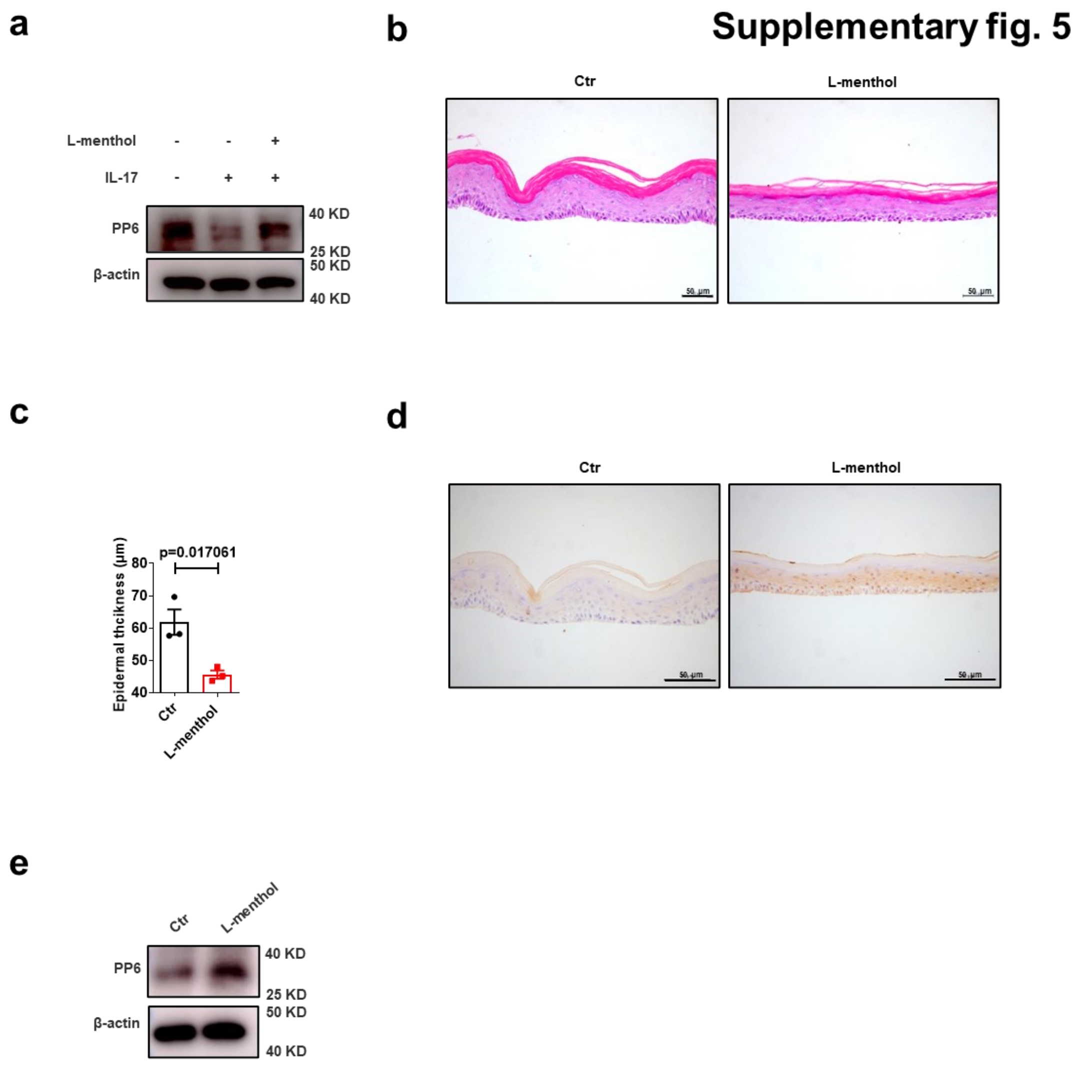

Supplementary fig. 5 L-menthol upregulates PP6 in IL-17-stimulated normal human keratinocytes and psoriasiform 3-D human skin equivalents.

a, Immunoblotting analysis of PP6 expression in NHEK cells with or without 10 μM L-menthol treatment for 12 h in the presence or absence of 200 ng/ml IL-17A. **b,** Representative H&E staining of the psoriasiform 3-D human skin equivalent treated with DMSO (Ctr) or 10 μM L-menthol. Scale bar, 50μm. **c,** Epidermal thickness of psoriasiform 3-D human skin equivalent treated with DMSO (Ctr) or 10 μM L-menthol (n=3). **d,** Representative immunohistochemical staining of PP6 in psoriasiform 3-D human skin equivalent treated with DMSO (Ctr) or 10 μM L-menthol. Scale bar, 50μm. **e,** Immunoblotting analysis of PP6 expression in psoriasiform 3-D human skin equivalent

treated with DMSO (Ctr) or 10 μM L-menthol. The data in (**a-e**) are representative of three independent experiments. Statistical analyses were performed by two-tailed Student's t test. All data are presented as mean values ± SEM. Specific p-values are indicated in the figure. Source data are provided as a Source Data file.

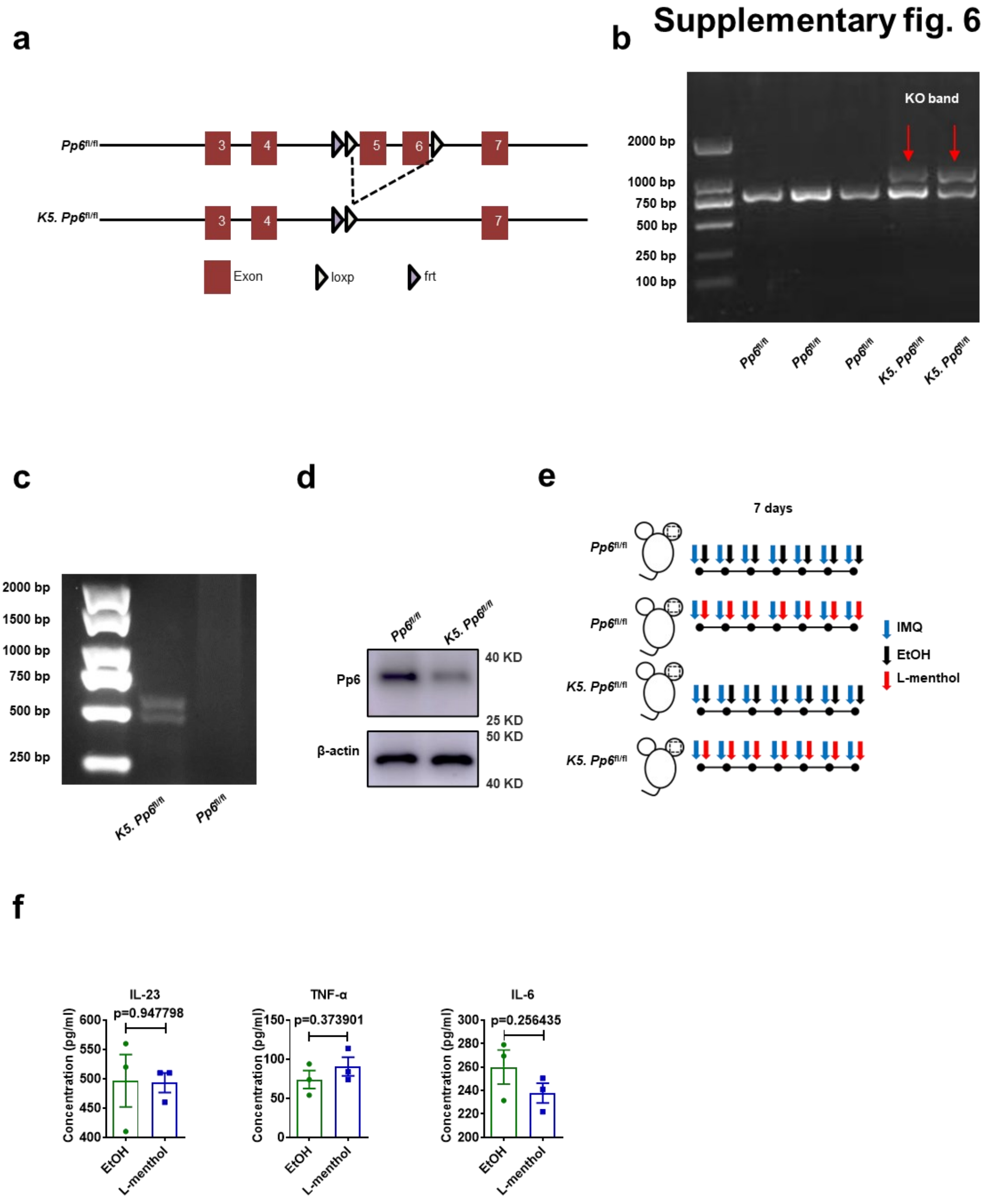

Supplementary fig. 6 L-menthol fails to decrease inflammatory cytokines in *K5. Pp6*$^{fl/fl}$ mice.

a, Schematic of *Pp6* deletion in *K5. Pp6*$^{fl/fl}$ mice. **b,** *Pp6* genotyping of the skin derived from *Pp6*$^{fl/fl}$ and *K5. Pp6*$^{fl/fl}$ mice. **c,** *K5* genotyping of the skin derived from *Pp6*$^{fl/fl}$ and *K5. Pp6*$^{fl/fl}$ mice. **d,** Immunoblotting analysis of Pp6 expression in the epidermis derived from *Pp6*$^{fl/fl}$ and *K5. Pp6*$^{fl/fl}$ mice. **e,** Schematic of IMQ-induced psoriasis mouse model and 50 μg/day L-menthol treatment in *Pp6*$^{fl/fl}$ mice or *K5. Pp6*$^{fl/fl}$ mice. **f,** Enzyme-linked immunosorbent assay (ELISA) for IL-23, TNF-

α and IL-6 in skin lesions derived from IMQ-induced *K5. Pp6*$^{fl/fl}$ mice with the treatment of EtOH or 50 μg/day L-menthol (n=3). The data in (**b-d, f**) are representative of three independent experiments. Statistical analyses were performed by two-tailed Student's t test. All data are presented as mean values ± SEM. Specific p-values are indicated in the figure. Source data are provided as a Source Data file.

Supplementary fig. 7

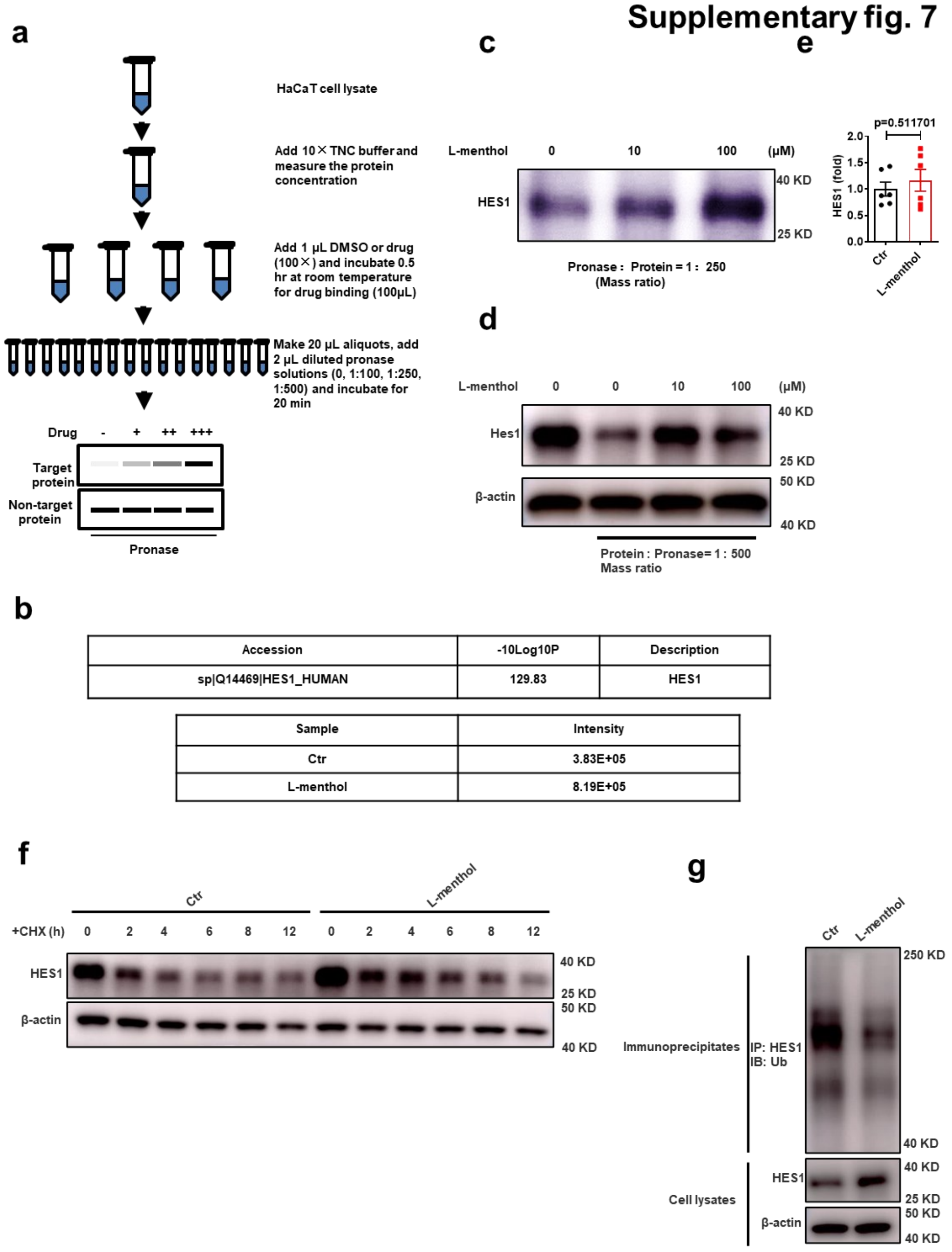

Supplementary fig. 7 Identification of the target for L-menthol in keratinocytes.

a, Experimental scheme of DARTS using HaCaT cell lysates. **b,** LC-MS/MS-based detection of HES1 abundance in HaCaT cell lysates with or without L-menthol subjected to pronase digestion. The significance score is calculated as the -10log10 of the significance testing p-value. Paired T-test is used for significance calculation. **c,** Immunoblotting analysis of HES1 expression in purified

protein HES1 with or without L-menthol subjected to pronase digestion. **d,** Immunoblotting analysis of HES1 expression in the epidermis derived from IMQ-induced mice with or without L-menthol subjected to pronase digestion. **e,** qPCR detection of HES1 in HaCaT cells treated with or without 10μM L-menthol for 12 h in the presence of 1μg/ml. Results are presented as the ratio of HES1 to the GAPDH, relative to that in control. **f,** Immunoblotting analysis of HaCaT cells treated with 1 μg/ml R848 and DMSO or 10 μM L-menthol along with 25 μg/ml cycloheximide for the indicated time. **g,** Immunoblotting of HES1-immunoprecipitated ubiquitin in HaCaT cells treated with DMSO (Ctr) or 10 μM L-menthol for 12 hours in the presence of 1 μg/ml R848. The data in (**c-g**) are representative of three independent experiments. Statistical analyses were performed by two-tailed Student's t test. All data are presented as mean values ± SEM. Specific p-values are indicated in the figure. Source data are provided as a Source Data file.

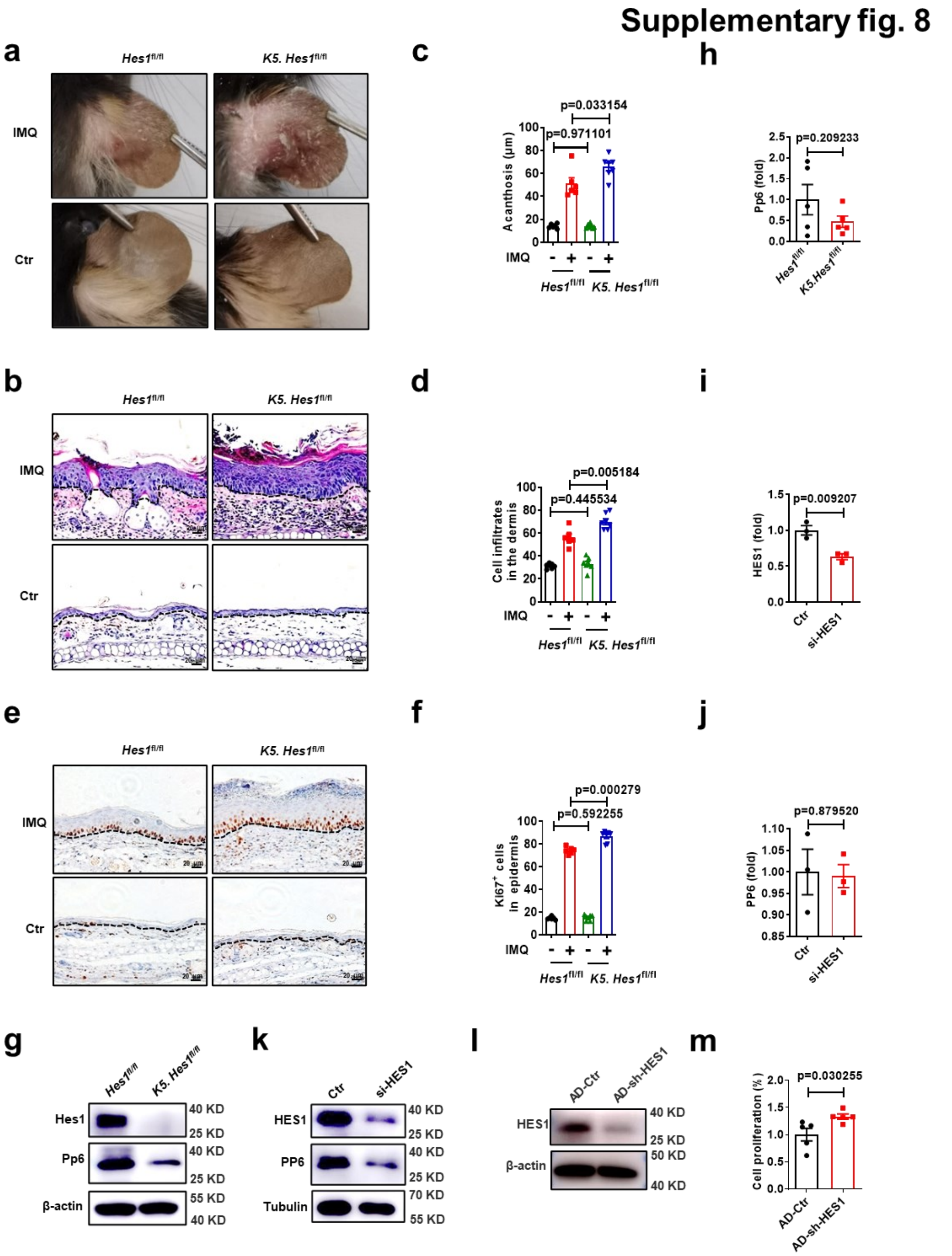

Supplementary fig. 8 Hes1 deficiency in keratinocytes decreases Pp6 and exacerbates IMQ-induced skin inflammation.

a, Representative photographs of the ears from *Hes1*$^{fl/fl}$ mice and *K5. Hes1*$^{fl/fl}$ mice with or without IMQ induction (n=6-7). **b,** Representative H&E staining of the ears treated as in (**a**) (n = 6-7). Scale

bar, 20 μm. **c-d,** Acanthosis (**c**) and dermal cellular infiltrates (**d**) of ears treated as in (**a**) (n=6-7). **e,** Representative immunohistochemical staining of Ki67 in ears treated as in (**a**) (n=6-7). Scale bar, 20 μm. **f,** Quantitation of $Ki67^+$ epidermal cells in ears treated as in (**a**) (n=6-7). **g,** Immunoblotting analysis of Hes1 and Pp6 expression in epidermis derived from $Hes1^{fl/fl}$ mice or $K5.\ Hes1^{fl/fl}$ mice. **h,** qPCR detection of Pp6 in epidermis derived from $Hes1^{fl/fl}$ mice or $K5.\ Hes1^{fl/fl}$ mice (n = 5). The data are presented as the ratio of Hes1 to GAPDH relative to that in epidermis from $Hes1^{fl/fl}$ mice. **i-j,** qPCR detection of HES1 (**i**) and PP6 (**j**) in HaCaT cells transfected scramble siRNA (Ctr) or with HES1 siRNA (si-HES1) (n = 3). The data are presented as the ratio of HES1 or PP6 to GAPDH relative to that in scramble-transfected HaCaT cells. **k,** HaCaT cells were transfected with scramble siRNA (Ctr) or with HES1 siRNA (si-HES1). Cell lysates were immunoblotted with anti-HES1, anti-PP6 or anti-Tubulin. **l,** Immunoblotting analysis of HES1 expression in NHEK cells 2 days after the infection of adeno virus. **m,** Cell proliferation of NHEK cells in (**l**). The data in (**a-m**) are representative of three independent experiments. Statistical analyses were performed by two-tailed Student's t test. All data are presented as mean values ± SEM. Specific p-values are indicated in the figure. Source data are provided as a Source Data file.

a **Supplementary fig. 9**

Protein information			Intensity	
Accession	-10Log10P	Description	Ctr	L-menthol
sp|Q01105|SET_HUMAN	47.79	SET	2.10E+05	UD
sp|O14965|AURKA_HUMAN	26.08	AURKA	UD	4.96E+04
sp|P78318|IGBP1_HUMAN	28.17	IGBP1	1.07E+04	8.44E+04
sp|Q14469|HES1_HUMAN	UD	HES1	UD	UD

b

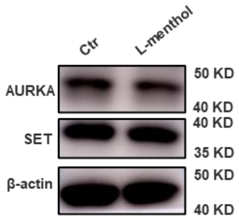

c

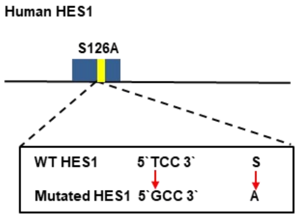

d

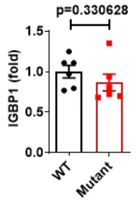

Supplementary fig. 9 The phosphorylation of S126 in HES1 is dispensable for the activation of IGBP1.

a, LC-MS/MS-based detection of PP6-immunoprecipitated SET, AURKA, IGBP1 and HES1 abundance in HaCaT cells treated with or without 10 μM L-menthol in the presence of 1 μg/ml R848. UD, undetected. The significance score is calculated as the -10log10 of the significance testing p-value. Paired T-test is used for significance calculation. **b,** Immunoblotting analysis of AURKA and SET expression in HaCaT cells with or without 10 μM L-menthol treatment for 12 h in the presence of 1 μg/ml R848. **c,** Schematic of kinase domain mutated (S126A) form of HES1. **d,** qPCR detection of IGBP1 in HaCaT cells transfected with wild-type HES1 (WT) or kinase domain mutated (S126A) form of HES1 (Mutant). The data are presented as the ratio of IGBP1 to GAPDH relative to that in WT (n = 6). The data in (**b, d**) are representative of three independent

experiments. Statistical analyses were performed by two-tailed Student's t test. All data are presented as mean values ± SEM. Specific p-values are indicated in the figure. Source data are provided as a Source Data file.

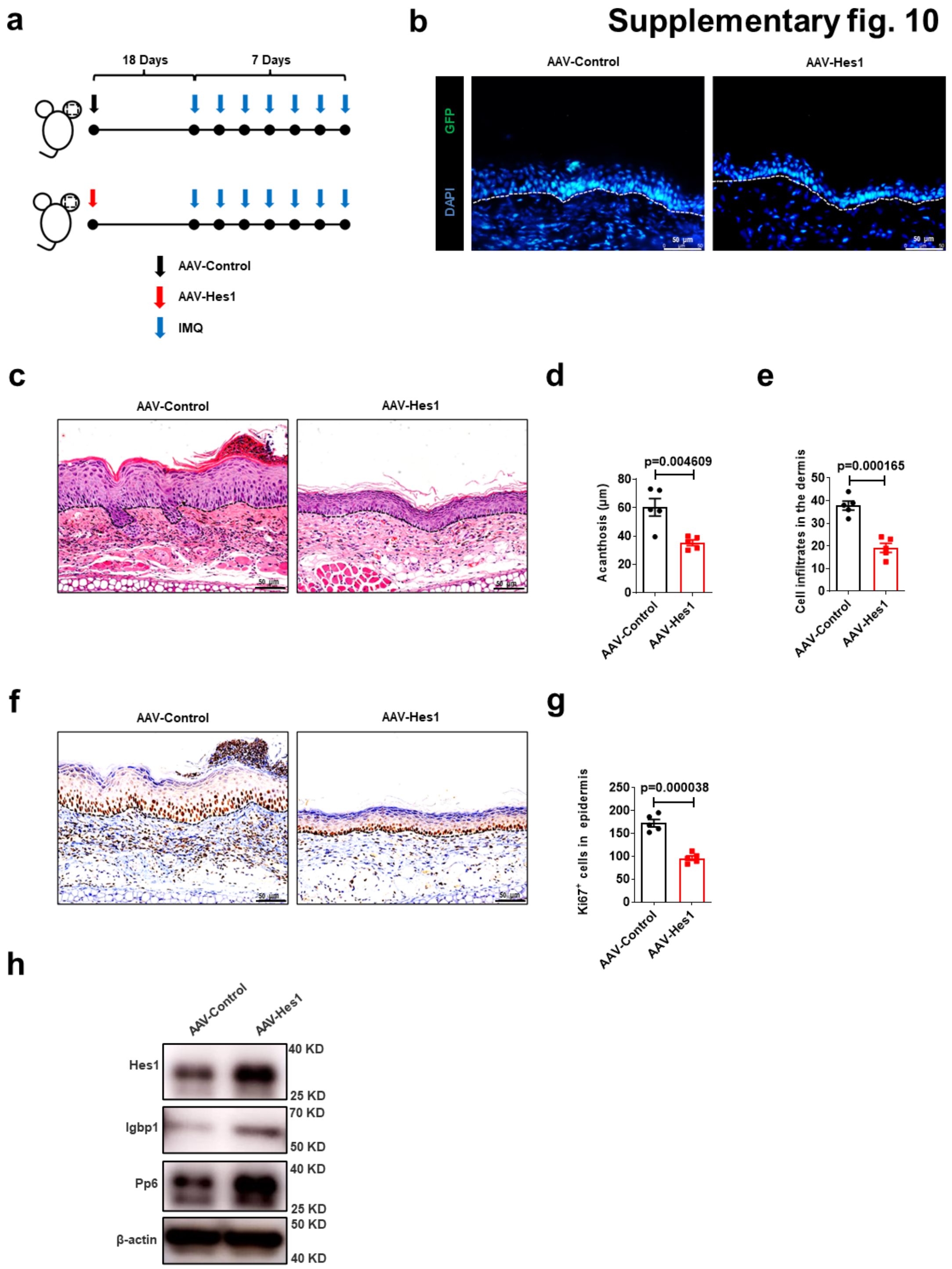

Supplementary fig. 10 Hes1-upregulation in keratinocytes relieves psoriasis like skin inflammation in mice.

a, Schematic of the subcutaneous injection of GFP-expressing adeno-associated virus (AAV) into the ear skin of IMQ-induced mice. **b,** Fluorescence images showing GFP signals of the injected skin

25 days after the injection of AAV. Scale bar, 50 μm. **c,** Representative H&E staining of the ears of IMQ-induced mice with the subcutaneous injection of AAV-Control or AAV-Hes1. Scale bar, 50 μm. **d-e,** Acanthosis (**d**) and dermal cellular infiltrates (**e**) of ears treated as in (**c**) (n=5). **f,** Representative immunohistochemical staining of Ki67 of ears treated as in (**c**). Scale bar, 50 μm. **g,** Quantitation of $Ki67^+$ epidermal cells in ears treated as in (**c**) (n=5). **h,** Immunoblotting of Hes1, Igbp1 and Pp6 expression in epidermis derived from ears treated as in (**c**) (n=5). The data in (**b-h**) are representative of three independent experiments. Statistical analyses were performed by two-tailed Student's t test. All data are presented as mean values ± SEM. Specific p-values are indicated in the figure. Source data are provided as a Source Data file.

Supplementary fig. 11

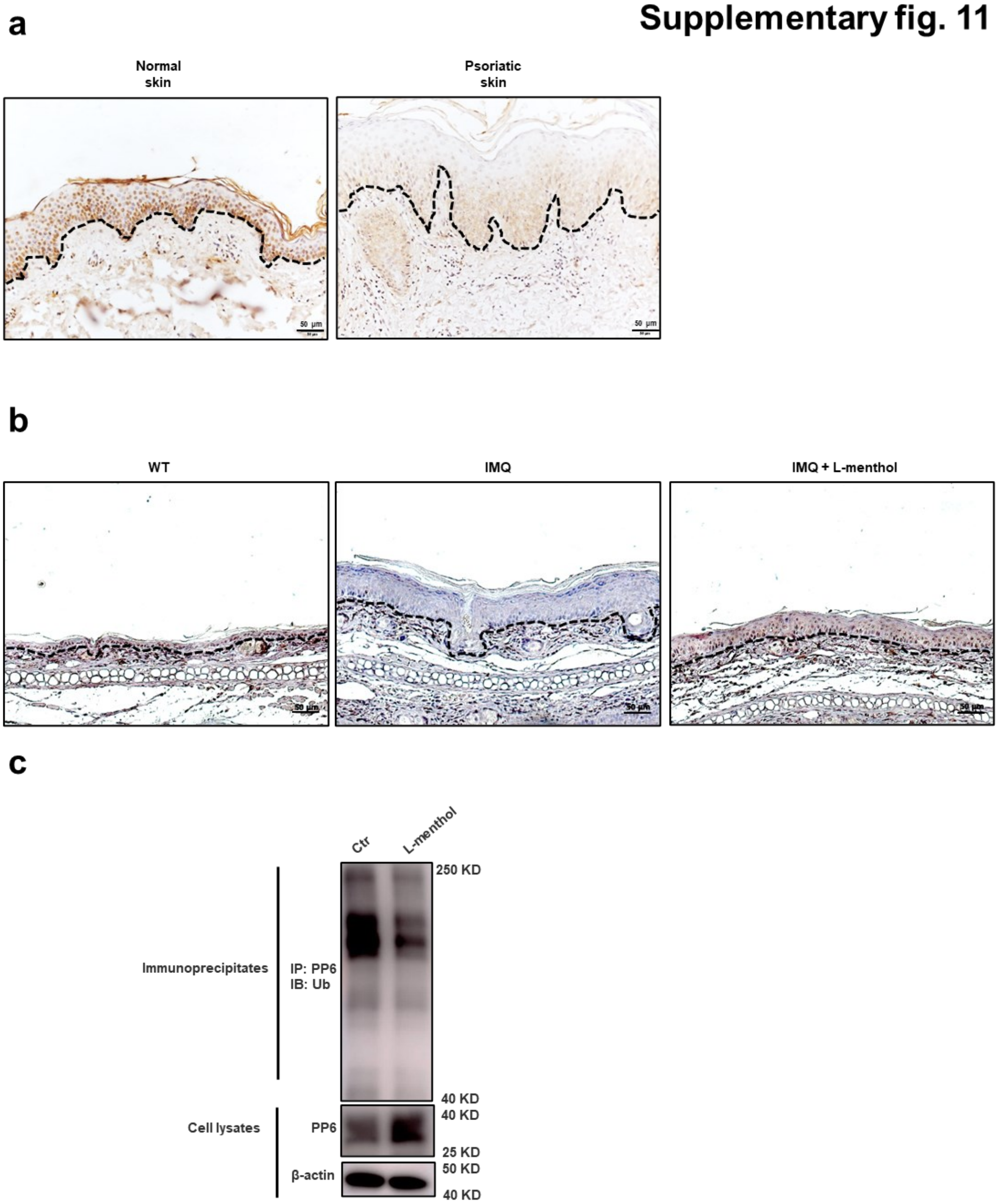

Supplementary fig. 11 L-menthol upregulates IGBP1 and decreases PP6-ubiquitination in inflamed keratinocytes.

a, Immunohistochemical staining of IGBP1 in skin sections derived from healthy or psoriatic skin. Scale bar, 50 μm. **b,** Representative immunohistochemical staining of Igbp1 in ears derived from WT mice, IMQ-induced mice and IMQ-induced mice with L-menthol treatment. Scale bar, 50 μm. **c,** Immunoblotting of PP6-immunoprecipitated ubiquitin in HaCaT cells treated with DMSO (Ctr)

or 10 μM L-menthol for 12 hours in the presence of 1 μg/ml R848. The data in (**a-c**) are representative of three independent experiments. Source data are provided as a Source Data file.

Supplementary table. Sequences of primers and oligonucleotides.

**Mouse primers**
Hes1 forward：5′-CCAACGGGGCCTTCGCTCACAG-3′
Hes1 reverse：5′-CGCCACGGTCTCCACAT-3′
Igbp1 forward：5′-ACCCTTCATCCTCACTCGGA-3′
Igbp1 reverse：5′-TTCCCCGGTCGGGTAATACT-3′
Pp6 forward：5′-TCTACCTGCTTTCCTTCGCTG-3′
Pp6 reverse：5′-GCCAGGGTCCAGATCCAGA-3′
Gapdh forward：5′-TGGCCTTCCGTGTTCCTAC-3′
Gapdh reverse：5′-GAGTTGCTGTTGAAGTCGCA-3′
**Human primers**
GAPDH forward: 5′-CTGGGCTACACTGAGCACC-3′
GAPDH reverse: 5′-AAGTGGTCGTTGAGGGCAATG-3′
PP6 forward: 5′-TGGAAATGCTAATGCCTGGAG-3′
PP6 reverse: 5′-ACCTGATTCCGTTCGATGGTTC-3′
HES1 forward: 5′-ATGGAGAAAAATTCCTCGTCCC-3′
HES1 reverse: 5′-TTCAGAGCATCCAAAATCAGTGT-3′
AURKA forward: 5′- CAGACTGGATACCGGGACC-3′
AURKA reverse: 5′-CTTCAGCACGTTTTTGCACTG-3′
PPP6R3 forward: 5′- GTAAAGCTCAGAACCGCAAACT-3′
PPP6R3 reverse: 5′-ATCTGGGAGACATCAGAAGTGA-3′
SET forward: 5′-AGCAAGAAGCGATTGAACACA-3′
SET reverse: 5′-TGGTTGGCGGAGTTTGTTATATT-3′
IGBP1 forward: 5′-GAGAGGCCTCCAGTGAAACC-3′
IGBP1 reverse: 5′-GAATTCCTCTGGTGCTGCCT-3′
**Primers for luciferase reporter assays**
Igbp1 promoter forward: 5′-CGGGGTACCAGACAGTGTGCCTGCTACTT-3′
Igbp1 promoter reverse: 5′- CCGCTCGAGCCACTCCCACGGTTTGAAGA-3′
Igbp1 mutant promoter forward: 5′-ACGGGTCGTAAGAGAGAGAGGGGTGAAGGGGGAA-3′
Igbp1 mutant promoter reverse: 5′-CCCTTCACCCCTCTCTCTCTTACGACCCGTAGGC-3′
**Small interfering RNA (siRNA) targeting human IGBP1 or HES1**
IGBP1 siRNA sense: GCAUCUCAAAGACAGGCUATT
IGBP1 siRNA anti-sense: UAGCCUGUCUUUGAGAUGCTT
HES1 siRNA sense: GGAUGCUCUGAAGAAAGAUTT
HES1 siRNA anti-sense: AUCUUUCUUCAGAGCAUCCTT
Negative control sense：UUCUCCGAACGUGUCACGUTT
Negative control anti-sense：ACGUGACACGUUCGGAGAATT
