# Supplementary material for: Targeting the transcription factor HES1 by L-menthol restores protein phosphatase 6 in keratinocytes in models of psoriasis
Source: Nat Commun. 2022 Dec 19;13:7815. doi: 10.1038/s41467-022-35565-y (PMC9763329; doi:10.1038/s41467-022-35565-y)
Supplement: Supplementary file 3 — Description of Additional Supplementary Files [file 41467_2022_35565_MOESM3_ESM.docx]

**Description of Additional Supplementary Files**

**Supplementary Data 1:** LC-MS/MS-based detection of protein abundance in HaCaT cell lysates with or without L-menthol subjected to pronase digestion. The significance score is calculated as the -10lg of the significance testing p-value. Paired T-test is used for significance calculation.

**Supplementary Data 2:** LC-MS/MS-based detection of PP6-immunoprecipitated protein abundance in HaCaT cells treated with or without 10 μM L-menthol in the presence of 1 μg/ml R848. The significance score is calculated as the -10lg of the significance testing p-value. Paired T-test is used for significance calculation.
