# Supplementary material for: Targeting the transcription factor HES1 by L-menthol restores protein phosphatase 6 in keratinocytes in models of psoriasis
Source: Nat Commun. 2022 Dec 19;13:7815. doi: 10.1038/s41467-022-35565-y (PMC9763329; doi:10.1038/s41467-022-35565-y)
Supplement: Supplementary file 6 — Reporting Summary [file 41467_2022_35565_MOESM6_ESM.pdf]

nature portfolio

Corresponding author(s): Honglin WANG

Last updated by author(s): Oct 25, 2022

Reporting Summary

Nature Portfolio wishes to improve the reproducibility of the work that we publish. This form provides structure for consistency and transparency in reporting. For further information on Nature Portfolio policies, see our Editorial Policies and the Editorial Policy Checklist.

Statistics

For all statistical analyses, confirm that the following items are present in the figure legend, table legend, main text, or Methods section.

n/a	Confirmed	
☐	☒	The exact sample size (n) for each experimental group/condition, given as a discrete number and unit of measurement
☐	☒	A statement on whether measurements were taken from distinct samples or whether the same sample was measured repeatedly
☐	☒	The statistical test(s) used AND whether they are one- or two-sided<br>*Only common tests should be described solely by name; describe more complex techniques in the Methods section.*
☒	☐	A description of all covariates tested
☐	☒	A description of any assumptions or corrections, such as tests of normality and adjustment for multiple comparisons
☐	☒	A full description of the statistical parameters including central tendency (e.g. means) or other basic estimates (e.g. regression coefficient) AND variation (e.g. standard deviation) or associated estimates of uncertainty (e.g. confidence intervals)
☐	☒	For null hypothesis testing, the test statistic (e.g. F, t, r) with confidence intervals, effect sizes, degrees of freedom and P value noted<br>*Give P values as exact values whenever suitable.*
☒	☐	For Bayesian analysis, information on the choice of priors and Markov chain Monte Carlo settings
☒	☐	For hierarchical and complex designs, identification of the appropriate level for tests and full reporting of outcomes
☒	☐	Estimates of effect sizes (e.g. Cohen's d, Pearson's r), indicating how they were calculated

Our web collection on statistics for biologists contains articles on many of the points above.

Software and code

Policy information about availability of computer code

Data collection	As described in Methods:<br>Histopathological image was captured using Axiovision software.<br>scRNA-seq data were processed using Cell Ranger (version 3.0)<br>MST data was collected by using Monolith NT.115 (NanoTemper Technologies).
Data analysis	The data were analyzed using GraphPad Prism 6.02, Monolith NT.115, Seurat package (Version 3.0), and Adobe Photoshop CS4.

For manuscripts utilizing custom algorithms or software that are central to the research but not yet described in published literature, software must be made available to editors and reviewers. We strongly encourage code deposition in a community repository (e.g. GitHub). See the Nature Portfolio guidelines for submitting code & software for further information.

Data

Policy information about availability of data

All manuscripts must include a data availability statement. This statement should provide the following information, where applicable:

- Accession codes, unique identifiers, or web links for publicly available datasets
- A description of any restrictions on data availability
- For clinical datasets or third party data, please ensure that the statement adheres to our policy

The single-cell RNA-sequencing data in this study have been deposited in the Genome Sequence Archive (GSA) with accession number HRA003418 (https://ngdc.cncb.ac.cn/gsa-human/browse/HRA003418). The mass spectrometry proteomics data have been deposited to the ProteomeXchange Consortium via the PRIDE partner repository with the dataset identifier PXD037335 (https://www.ebi.ac.uk/pride/archive/projects/PXD037335) and PXD037336 (https://www.ebi.ac.uk/pride/archive/projects/PXD037336). The other data supporting the findings of this study are available within the paper or the supplementary materials. Source data are provided with this paper.

Human research participants

Policy information about studies involving human research participants and Sex and Gender in Research.

Reporting on sex and gender	Sex was not considered in study designed.
Population characteristics	The age of human research participants is between 25 to 59. Anatomic sites include trunk, limbs, and eyelid. Psoriasis patient information is provided in the source data. The duration of disease of three psoriasis patients is 1 months, 5 months and 2 years respectively. They haven't been treated before (systemic or local treatment).
Recruitment	Psoriatic skin samples were obtained by punch biopsy from patients who were under local lidocaine anesthesia. Normal adult human skin specimens were taken from healthy donors who were undergoing plastic surgery. All participants provided written informed consent. Three male psoriasis patients without any treatment are randomly recruited.
Ethics oversight	The study was performed in accordance with the principles of the Declaration of Helsinki and approved by the Research Ethics Boards of Xiangya Hospital of Central South University and Shanghai General Hospital (No. 201311392 and No. 2018KY239)

Note that full information on the approval of the study protocol must also be provided in the manuscript.

Field-specific reporting

Please select the one below that is the best fit for your research. If you are not sure, read the appropriate sections before making your selection.

☒ Life sciences ☐ Behavioural & social sciences ☐ Ecological, evolutionary & environmental sciences

For a reference copy of the document with all sections, see nature.com/documents/nr-reporting-summary-flat.pdf

Life sciences study design

All studies must disclose on these points even when the disclosure is negative.

Sample size	In vitro and in vivo experiments were done at least three times, either in duplicate or triplicate. In general, at least three mice were used per group per experiment. Sample size was largely based on availability of germ-free mice and age matched controls. Besides, at least triplicate wells of cells were used per group per experiment. Sample size was determined based on the set-ups in previous reports to ensure adequate power.<br>1.Yan S, Xu Z, Lou F, et al. NF-κB-induced microRNA-31 promotes epidermal hyperplasia by repressing protein phosphatase 6 in psoriasis. Nat Commun. 2015;6:7652.<br>2.Lou F, Sun Y, Xu Z, et al. Excessive Polyamine Generation in Keratinocytes Promotes Self-RNA Sensing by Dendritic Cells in Psoriasis. Immunity. 2020;53(1):204-216.e10.
Data exclusions	No data were excluded from analyses.
Replication	All experiments were performed with independent replicates as described in the figure legends
Randomization	Cells were randomly allocated into groups. Mice with comparable age and weight were randomly selected from housing cages and then assigned in groups with no bias for further treatment. Human samples were randomized within healthy and psoriasis groups and assayed together for single cell RNA sequencing and immunohistochemical staining.
Blinding	Investigators were blinded to group allocation during data collection and analysis.

Reporting for specific materials, systems and methods

We require information from authors about some types of materials, experimental systems and methods used in many studies. Here, indicate whether each material, system or method listed is relevant to your study. If you are not sure if a list item applies to your research, read the appropriate section before selecting a response.

Materials & experimental systems

n/a	Involved in the study
☐	☒ Antibodies
☐	☒ Eukaryotic cell lines
☒	☐ Palaeontology and archaeology
☐	☒ Animals and other organisms
☒	☐ Clinical data
☒	☐ Dual use research of concern

Methods

n/a	Involved in the study
☒	☐ ChIP-seq
☒	☐ Flow cytometry
☒	☐ MRI-based neuroimaging

Antibodies

Antibodies used

Anti-mouse Ki67 (Servicebio cat. GB121141, 1:500 dilution), anti-PPP6C (Merck Millipore cat. 07-1224, 1:1000 dilution), anti-β-actin (Proteintech cat. 60008-1-Ig, 1:4000 dilution), anti-HES1 (Cell Signaling Technology cat. 11988S, 1:1000 dilution), anti-IGBP1 (Abcam cat. ab170911, 1:1000 dilution), anti-alpha Tubulin (Proteintech cat. 66031-1-Ig, 1:2000 dilution), anti-CHIP (Abcam cat. ab134064, 1:1000 dilution), anti-HA-Tag (Proteintech cat. 66006-2-Ig, 1:10000 dilution), anti-SET (Abcam cat. 181990, 1:1000 dilution), anti-AURKA (Abcam cat. 247643, 1:1000 dilution), anti-Ubiquitin (Cell Signaling Technology cat.3936S, 1:1000 dilution), anti-TLR7 (Novus cat. NBP2-27332, 1:100 dilution), anti-C/EBP-β (Abcam cat. 32358, 1:1000 dilution), anti-p-C/EBP-β (Thr188) (Cell Signaling Technology cat. 3084, 1:1000 dilution), HRP-labeled goat anti-mouse IgG (H+L) (Beyotime cat. A0216, 1:1000 dilution), HRP-labeled goat anti-rabbit IgG (H+L) (Beyotime cat. A0208, 1:1000 dilution), anti-PPP6C (Abcam cat. 131335, 1:50 dilution) and anti-rabbit IgG (Abcam cat. 172730, 1:50 dilution) were used as antibodies.

Validation

All antibodies were validated for use in immunohistochemistry and/or immunoprecipitation and/or immunoblotting. All validation statements can be found on the respective antibody website:
1.Anti-mouse Ki67: https://www.servicebio.cn/goodsdetail?id=6801
2.anti-PPP6C: https://www.merckmillipore.com/CN/zh/product/Anti-PP6C-Antibody,MM_NF-07-1224
3. anti-β-actin: https://www.ptglab.co.jp/products/ACTB-Antibody-60008-1-Ig.htm
4. anti-HES1: https://www.cellsignal.cn/products/primary-antibodies/hes1-d6p2u-rabbit-mab/11988
5. anti-IGBP1: https://www.abcam.com/igbp1-antibody-epr12315-ab170911.html
6. anti-alpha Tubulin: https://www.ptglab.co.jp/products/tubulin-Alpha-Antibody-66031-1-Ig.htm
7. anti-CHIP: https://www.abcam.com/stub1chip-antibody-epr4447-ab134064.html
8. anti-HA-Tag: https://www.ptglab.co.jp/products/HA-Tag-Antibody-66006-2-Ig.htm
9. anti-SET: https://www.abcam.com/settaf-i-antibody-epr12973-ab181990.html
10. anti-AURKA: https://www.abcam.com/aurora-a-antibody-epr5026-bsa-and-azide-free-ab247643.html
11. anti-Ubiquitin: https://www.cellsignal.com/products/primary-antibodies/ubiquitin-p4d1-mouse-mab/3936
12. anti-TLR7: https://www.novusbio.com/products/tlr7-antibody-4g6_nbp2-27332
13. anti-C/EBP-β: https://www.abcam.com/cebp-beta-antibody-e299-c-terminal-ab32358.html
14. anti-p-C/EBP-β (Thr188): https://www.cellsignal.com/products/primary-antibodies/phospho-c-ebpb-thr235-antibody/3084
15. HRP-labeled goat anti-mouse IgG (H+L): https://www.beyotime.com/product/A0216.htm
16. HRP-labeled goat anti-rabbit IgG (H+L): https://www.beyotime.com/product/A0208.htm
17. anti-PPP6C: https://www.abcam.cn/ppp6cppv-antibody-epr8764-ab131335.html
18. anti-rabbit IgG: https://www.abcam.cn/rabbit-igg-monoclonal-epr25a-isotype-control-ab172730.html

Eukaryotic cell lines

Policy information about cell lines and Sex and Gender in Research

Cell line source(s)

HaCaT cells (Mxbio, cat. MXC138)
Primary normal human epidermal keratinocyte (NHEK) (Lifeline Cell Technology, cat. FC-0025.)

Authentication

Quality testing for primary normal human epidermal keratinocytes (NHEK) is provided on the website of Lifeline Cell Technology (https://www.lifelinecelltech.com/shop/tissue-type/skin-tissue/human-epidermal-keratinocytes-adult-fc-0025/).

Quality testing for HaCaT is provided on the website of Mxbio (http://www.mxbio.cn/product.html?keywords=mxc138).

Mycoplasma contamination

Cell lines tested negative for mycoplasma contamination.

Commonly misidentified lines
(See ICLAC register)

No commonly misidentified cell lines were used in the study.

Animals and other research organisms

Policy information about studies involving animals; ARRIVE guidelines recommended for reporting animal research, and Sex and Gender in Research

Laboratory animals	C57BL/6 mice were purchased from Shanghai SLAC Laboratory Animal Co., Ltd. (Shanghai, China).<br>Mice with loxP-flanked Pp6 alleles were provided by Dr. Wufan Tao (State Key Laboratory of Genetic Engineering and Institute of Developmental Biology and Molecular Medicine, Fudan University, Shanghai, China)<br>Keratin 5-Cre transgenic mice were provided by Dr. Xiao Yang (State Key Laboratory of Proteomics, Genetic Laboratory of Development and Disease, Institute of Biotechnology, Beijing, China)<br>mice with loxP-flanked Hes1 alleles were were originally obtained from R. Kageyama (Kyoto University, Kyoto, Japan)<br>All mice used in this study were 6–10 weeks old.<br>The catalogue number of all diet is cat.1010086 (Jiangsu Xietong Pharmaceutical Bio-engineering Co., Ltd, China).<br>Mice were used for all of the experiments in accordance with the National Institutes of Health Guide for the Care and Use of Laboratory Animals with the approval (SYXK-2003-0026) of the Scientific Investigation Board of Shanghai Jiao Tong University School of Medicine in Shanghai, China.<br>Mice were housed in cages with five mice per cage and kept on in a regular 12-h:12-h light: dark cycle. The temperature was 22±1 degree Celsius and humidity was 40-70 %.
Wild animals	The study did not involve wild animals.
Reporting on sex	For the IMQ-induced mouse model of psoriasis, male C57BL/6 mice (7 weeks of age) were maintained under SPF conditions. For the experiment involved with Pp6 flox/flox mice, Pp6 flox/flox K5 cre mice, Hes1 flox/flox mice and Hes1 flox/flox K5 cre mice, sex was not considered in study design.
Field-collected samples	No field-collected samples were used in this study.
Ethics oversight	Mice were used for all of the experiments in accordance with the National Institutes of Health Guide for the Care and Use of Laboratory Animals with the approval (SYXK-2003-0026) of the Scientific Investigation Board of Shanghai Jiao Tong University School of Medicine in Shanghai, China.

Note that full information on the approval of the study protocol must also be provided in the manuscript.
